# Fuelling walking and cycling: human powered locomotion is associated with non-negligible greenhouse gas emissions

**DOI:** 10.1038/s41598-020-66170-y

**Published:** 2020-06-08

**Authors:** Anja Mizdrak, Linda J Cobiac, Christine L Cleghorn, Alistair Woodward, Tony Blakely

**Affiliations:** 1 Burden of Disease Epidemiology, Equity and Cost-Effectiveness Programme (BODE 3), Department of Public Health, University of Otago (Wellington), 23 Mein Street, Newtown, Wellington, New Zealand; 2 Centre for Population Approaches to Non-Communicable Disease Prevention, Nuffield Department of Population Health, University of Oxford, Old Road Campus, Oxford, United Kingdom; 3 Epidemiology and Biostatistics, Department of Population Health, University of Auckland, Auckland, New Zealand; 4 Melbourne School of Population and Global Health, University of Melbourne, Melbourne, Australia

**Keywords:** Environmental impact, Climate-change policy, Risk factors

## Abstract

Reducing motorized transport and increasing active transport (i.e. transport by walking, cycling and other active modes) may reduce greenhouse gas (GHG) emissions and improve health. But, active modes of transport are not zero emitters. We aimed to quantify GHG emissions from food production required to fuel extra physical activity for walking and cycling. We estimate the emissions (in kgCO_2_e) per kilometre travelled for walking and cycling from energy intake required to compensate for increased energy expenditure, and data on food-related GHG emissions. We assume that persons who shift from passive modes of transport (e.g. driving) have increased energy expenditure that may be compensated with increased food consumption. The GHG emissions associated with food intake required to fuel a kilometre of walking range between 0.05 kgCO_2_e/km in the least economically developed countries to 0.26 kgCO_2_e/km in the most economically developed countries. Emissions for cycling are approximately half those of walking. Emissions from food required for walking and cycling are not negligible in economically developed countries which have high dietary-related emissions. There is high uncertainty about the actual emissions associated with walking and cycling, and high variability based on country economic development. Our study highlights the need to consider emissions from other sectors when estimating net-emissions impacts from transport interventions.

## Introduction

Globally, transport is responsible for about a quarter of total energy related greenhouse gas (GHG) emissions, and transport emissions have increased at a faster rate than any other energy end-use sector^1^. Light vehicles account for the use of half of all transport energy and around 40% is used in urban transport^1^.

The transport sector also influence population health – directly through road injury and indirectly through conditions caused by air pollution and exposure to noise^2^. There are also substantial indirect effects, through the influence of transport systems on patterns of physical activity^2^. Reducing motorized transport and increasing active transport (i.e. active modes of transport such as walking and cycling) may both reduce GHG emissions and improve population health. Recent research has quantified the co-benefits of increasing active transport in a range of settings – from city-level^3^ to international assessments^4^.

Increasing active transport appears to increase total physical activity^5^, leading, by a variety of mechanisms to a reduction in risk of non-communicable diseases and improvements in mental health^6,7^. Small increases in walking and cycling would be expected to result in weight loss due to the increased energy expenditure required (relative to sedentary time), unless energy intake is increased to compensate for the additional expenditure. However, reviews of the effect of active transport interventions on body weight are inconclusive^8,9^. Those who walk and cycle are reported to be lighter than people who travel by car, but most studies have been cross-sectional, leaving doubts about whether people shifting to active transport lose weight as a result or are lower weight for other reasons (i.e. confounding). Few longitudinal studies have tackled this question. Mode shift from passive to active forms of transport has been associated with reductions in body mass index (BMI)^5,10,11^. However, these longitudinal studies do not include comprehensive assessments of all changes in individual circumstances that may confound the observed relationship. Whilst some capture major life events (e.g. moving house, new job), none capture changes in dietary patterns or changes in neighbourhoods – factors that could influence both active transport and dietary patterns. Therefore, studies of active transport do not currently provide us with definitive information on the extent or nature of compensatory food intake in response to increased walking and cycling.

More broadly, the evidence on the nature and extent of compensatory food intake in response to physical activity more generally (and not just through active transport) is limited by the lack of adequately powered trials of sufficient duration that have adequately measured both energy expenditure and energy intake^12^. Although meta-analysis of laboratory studies show that individuals tend not to compensate for increased energy expenditure from exercise in the immediate hours after exercise^13^, the extent and nature of compensation for increased energy expenditure from physical activity in the longer term is not well established^12^.

For the purpose of this analysis, we assume that individuals who take up walking and cycling, or increase the amount by which they walk and cycle, would need to consume more food if they were to compensate for the increase in energy expenditure. The emissions impacts of this potential increase in food intake have not been considered in studies estimating the impact of increased active travel on GHG emissions. If we are to accurately estimate the net GHG emissions impacts of changing travel patterns, then we should include the environmental consequences of food needed to drive human-powered locomotion.

Berners-Lee (2010) estimated that a mile cycled (in the United Kingdom (UK)) generates between 65 gCO_2_e and 2,800 gCO_2_e depending on what the journey was powered by (bananas or air-freighted asparagus)^14^. The notion that energy expended from a cycle ride may be substituted directly by air-freighted asparagus is far-fetched, but underlines the point that there is a high carbon cost of modern food systems.

We now have data on the dietary GHG emissions associated with entire diets and not just individual food commodities. Estimates of dietary GHG emissions for adults range widely from around 100 gCO_2_e/100 kcal in China^15^ to 800 gCO_2_e/100 kcal in Australia^16,17^. These estimates of average GHG emissions associated with food intake allow us to build a more complete picture of the potential emissions associated with different active transport modes.

In this paper, we estimate the magnitude of emissions associated with fuel (in terms of food) required for walking and cycling. By combining information on energy expenditure from active transport and dietary patterns, we are able to estimate emissions required to travel a kilometre by walking and cycling. The emissions associated with energy required to fuel walking and cycling may not represent the actual emissions associated with active transport in real world settings where additional energy expenditure is only partially compensated with increased energy intake. Therefore, we explore the likely magnitude of emissions in a real-world setting with partial compensation of energy intake, using estimates from a UK cohort study.

## Results

**Figure 1:**
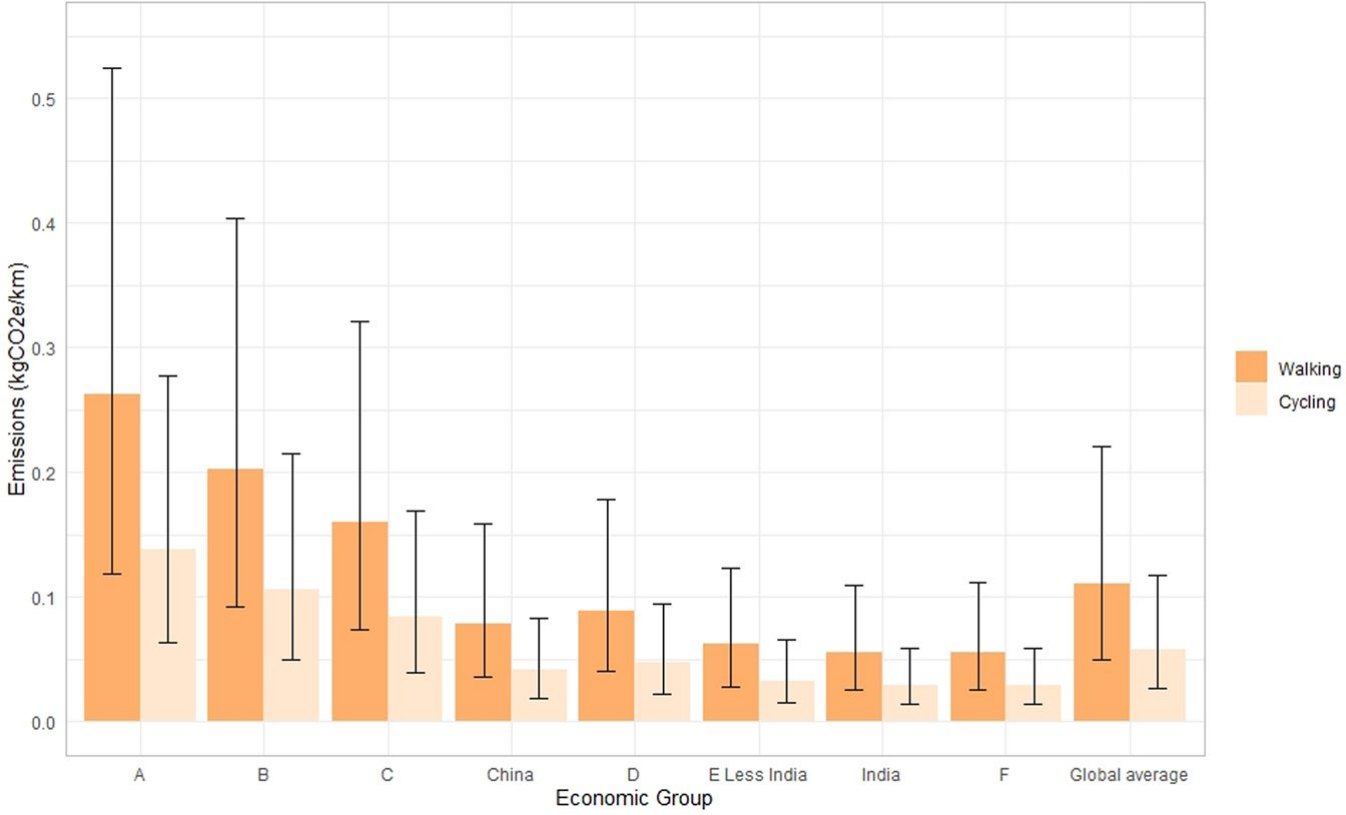
Emissions associated with travelling a kilometre, by walking, and cycling, assuming full energy compensation for countries grouped by level of economic development (from most economically developed (A) to least economically developed (F)).

Using estimates of energy availability^18^ (i.e. the energy available to be consumed, see Supplementary Table 1), we estimate that the additional energy expenditure required to travel 1 km ranged from 48 to 76 kcal for walking and 25 to 40 kcal for cycling (Supplementary Table 2). Based on the average global diet^18^, emissions required for walking are up to 0.11 kgCO2e/km (95% uncertainty interval (UI) 0.05 to 0.22)) and up to 0.14 kgCO_2_e/km (95% UI 0.06 to 0.28)) for cycling when energy expenditure is fully compensated with increased energy intake (Fig. 1). There is large variation between countries, in the most economically developed, emissions associated with energy required for walking are 2.4 times the global average.

### Estimated compensation of energy intake

We estimate that 57% of additional energy expenditure was compensated with additional food intake in the cohort study reported by Martin *et al*. (2015), with a range of 19% to 96% based on the confidence interval around the reported change in BMI. In the most economically developed countries (i.e. the group that includes the UK), 57% compensation would equate to net emissions of 0.15 kgCO_2_e/km for walking and 0.08 kgCO_2_e/km for cycling; 19% compensation equates to 0.05 kgCO_2_e/km for walking and 0.03 kgCO_2_e/km for cycling; 96% compensation equates to 0.25 kgCO_2_e/km for walking and 0.13 kgCO_2_e/km for cycling. This provides a potential range for the actual emissions impacts of walking and cycling in the most economically developed countries.

## Discussion

We estimate that walking and cycling an additional kilometre may result in GHG emissions up to 0.26 (95% UI 0.12 to 0.53) and 0.14 kgCO_2_e/km (95% UI 0.06 to 0.28)), respectively, when additional energy expenditure is fully compensated with increased food intake in the most economically developed countries. Our current best estimate for the most economically developed countries suggests that the actual emissions associated with walking and cycling will reflect partial compensation of energy intake - from 19% to 96% of the required energy intake. There is wide variability in emissions required to compensate for walking and cycling between countries, representing nearly a 5-fold difference between the most and least economically developed countries.

IPCC estimates that direct emissions from cars range from 0.08–0.21 kgCO_2_/km^1^, with a similar range of estimates in a more recent ‘well-to-wheel’ life cycle assessment (in the range of 0.15 kg/km to 0.26 kg/km^19–21^). Factors such as the composition of the vehicle fleet (age, manufacturer, size, etc), fuel type and where it is obtained from, driving behaviour, and road patterns are relevant. When comparing vehicle emissions with our estimates for walking and cycling, it is important to remember differences in the sources of emissions. Vehicular emissions are primarily drawn from non-renewable stocks, whereas estimates of dietary emissions include both renewable and non-renewable sources. Nevertheless, our estimates suggest that the net emissions associated with the ‘fuel’ required for driving, walking, and cycling may be comparable in some settings. Our intention was to provide an estimate of emissions required for walking and cycling, and explore the likely extent of compensatory intake. Our study demonstrates that overall assessments of the emissions impact of transport interventions should consider emissions impacts associated with diets. Taking account of walking and cycling emissions may suggest that car share schemes could have a bigger positive emissions impact than increasing walking, and (all else being equal) interventions that decrease vehicle use through increased cycling will have greater emissions benefits than those that increase walking.

Active transport has many advantages: more pleasant urban living, reduced air pollution, reduced non-communicable disease. But, to maximise the effect on GHG emissions achieved by increasing active transport, we need to address dietary patterns. Emissions associated with active transport will be lower if walking and cycling are powered by with low carbon dietary options, and/or associated with less than full compensation thereby resulting in lower obesity rates.

The GHG emissions associated with many modern diets can be reduced without health penalties. First, the way we as a society use energy to produce food can be altered. Like transport, agriculture is responsible for a substantial proportion of emissions, up to one-third of all anthropogenic GHG emissions by some estimates^22^. Second, given emissions associated with different food groups (given current food systems) range widely - from 0.02 (sugar, legumes) to 5.6 gCO_2_e/kcal (ruminant meats) in one global study^18^, consumer switching to less energy intense foods could reduce dietary emissions by up to 70–80%^22^. Third, food waste matters: the emissions footprint of the edible food that is lost or wasted each year is 3.5 GtCO2e/year^23^, equivalent to 50% of all transport emissions^1^. In high-income countries reductions in emissions are largely proportional to the magnitude of meat and dairy reduction^22^. That is, in order to reduce GHG emissions we need to encourage changes in what we eat as well as reducing motorized vehicle use.

At present, the magnitude of the impact of increased active transport on obesity is unclear and is likely to vary by setting and intervention. Quantifying the extent and nature of compensatory energy intake is needed to establish the health impact and GHG emissions impacts associated with active travel. Our results demonstrate that even in the countries with high dietary emissions, the emissions associated with walking and cycling may be considerably lower than the emissions associated with driving if individuals only partially compensate for the increased energy expenditure.

We estimate the compensation of additional energy expenditure with increased food intake ranged from 19% to 96% in one UK study that reported a BMI reduction with increased active transport^10^. For walking, this would translate to a range in diet-related emissions between 0.03 to 0.13 kgCO_2_e/km, equivalent to 12% to 63% of the emissions associated with the fuel for an average size car in the UK (0.21 kgCO_2_e/km^24^).Future research is needed to establish how compensatory behaviours vary between settings and across different interventions. For example, we would hypothesise that a media campaign that encourages active transport as a way to lose weight may be associated with lower levels of compensatory food intake and thereby may achieve greater population level reductions in obesity than an equivalent campaign encouraging active transport to reduce congestion.

To our knowledge, this is the first international estimate of GHG emissions associated with food intake required per kilometre travelled by active transport. Our results demonstrate the importance of including emissions associated with food intake when estimating the net GHG emissions impacts of interventions to increase active transport.

Our estimates are indicative of general patterns (i.e. that emissions required to fuel walking may be similar to emissions associated with fuelling a car when diets are highly carbon-intensive) but merit further consideration in more specific contexts. For example, how the emissions of walking or cycling compare to those of driving for specific trips in specific cities.

We limited our scope to emissions associated with the fuel required to walk and cycle a kilometre and do not include embodied emissions (e.g. emissions associated with the manufacture of cars or the construction and maintenance of transport systems). For interventions that switch selected (car) trips to walking or cycling, fuel-vs-fuel comparison may be appropriate, noting that the energy required to obtain and transport the fuel should be included (i.e. a well-to-wheels approach). Ideally, assessments of specific interventions should incorporate a wider view that considers the fuel-vs-fuel trade-offs, embodied emissions, and broader impacts (e.g. health and social costs of emissions), but adopting a wider view is a demanding and complex undertaking.

We use a single, global study as the source of both dietary emissions and energy availability^18^. As the data reflect energy availability, it is not subject to individual-level under-reporting present in survey data that would (falsely) deflate estimates of excess energy required for walking and cycling. Whilst estimates of energy availability will result in an overestimate of the energy intake required, they more accurately reflect the emissions associated with additional energy intake as they account for the amount of food lost and wasted prior to consumption. This means that our estimates implicitly account for food waste, assuming that food waste patterns do not change under increased active travel.

The estimates of dietary GHG emissions used are based on grouping countries at similar levels of economic development, using a consistent methodology. However, these results may mask variation in the dietary greenhouse gas emissions between countries with similar levels of economic development. This means that the true variation in emissions associated with walking and cycling is likely to be wider than represented here.

We explore the sensitivity of our results to assumptions around compensation of energy intake by modelling BMI changes and estimated emissions per km for a population based on a UK cohort study. The UK study was selected as it was the only longitudinal study we identified that presented sufficient data to estimate the extent of compensatory energy behaviour. However, our estimates of compensatory behaviour are based on aggregate results and not an analysis conducted at the individual level. As neither diet nor energy intake were assessed in the study, the observed change in weight may easily be confounded by other lifestyle changes accompanying transport mode shift (e.g. change in diet, income, or family circumstance). The reported change in BMI corresponds to a wide range in compensatory energy intake that is likely to be highly heterogeneous across individuals. The sensitivity of emissions estimates to compensatory behaviours in our example clearly demonstrates the need for better quantification of BMI changes and compensatory behaviour in the context of active transport.

If increased energy expenditure from active transport was not fully compensated, then both the food-related emissions associated with active transport will be lower and a (modest) contribution will be made to lowering obesity rates. Given the high impact of obesity on healthcare systems, and the high emissions associated with healthcare (e.g. 10% of total emissions in the USA^25^), the indirect benefits of active transport (such as through reduced health system burdens) onto emissions may be substantial, and warrant further research.

## Conclusions

Emissions of food required per kilometre of walking are not negligible in economically developed countries which have high dietary-related emissions. There is high uncertainty about the emissions associated with walking and cycling, and variability correlated with economic development. Our study highlights the need to consider emissions from other sectors when estimating net-emissions impacts from transport interventions.

## Methods

We calculated GHG emissions required to power a kilometre of walking and cycling for countries at different levels of economic development, as well as a global average using estimates of energy availability and dietary greenhouse gas emissions from a single global study^18^. First, we estimated the additional energy intake that would be required for travelling by walking and cycling relative to average daily activity. We then calculate the GHG emissions associated with compensating for the additional energy expenditure using estimates of the emissions per calorie associated with current dietary patterns. Finally, we explore the GHG emissions and BMI impacts associated with partial compensation of energy expenditure.

### Estimating excess energy expenditure

First, we calculated average energy expenditure in calories per minute by dividing estimates of daily calorie availability by 1,440 (the total number of minutes in a day). We assume that daily calorie availability data reflects energy intakes under current levels of energy expenditure (i.e. resting energy expenditure plus activity associated with daily life – hereafter referred to as average daily life), and the fact that some food is wasted prior to consumption^23^.

Next, we estimated the additional energy that would be required for a minute of travel by different modes of transport using MET (metabolic equivalent of task) values from the Compendium of Physical Activities^26^. A MET is the ratio of work metabolic rate to a standard resting metabolic rate, where one MET is equivalent to sitting quietly^26^. We estimated a MET value for average daily life of 1.5 to reflect average daily energy expenditure across a range of activities associated with daily living (e.g. sleep, occupation, personal care, housework). This is to reflect that current energy intakes represent intakes associated with average daily energy expenditure and not resting or basal rates of energy expenditure.

Walking was assigned a MET value of 3 (“walking, 2.5 mph, level, firm surface”), cycling a MET value of 4 (“bicycling, leisure, 5.5 mph”). Excess energy expenditure (i.e. excess relative to average daily life) was calculated by multiplying current energy intake (per min) by the ratio of the MET value of the walking and cycling relative to average daily life. We multiplied estimated excess energy expenditure by the time taken to walk and cycle a kilometre to give an estimate of per kilometre travelled. Estimates of the speed of walking and cycling were chosen to match with the selected MET values (i.e. 2.5 mph and 5.5 mph for walking and cycling respectively). Supplementary Table S2 presents the estimated excess energy expenditure in each economic group.

### Emissions per calorie

We used data on dietary GHG emissions for current dietary patterns to estimate emissions per 100 kcal. Dietary GHG estimates were taken from a study that use life-cycle assessment emissions data to calculate annual per capita GHG emissions from food production for the 2009 global average diet, and for groups of countries with similar levels of economic development^18^. The original analysis captured per capita dietary demand across different food groups including cereals, sugar, oil, fruits, vegetables, dairy and eggs, fish, and livestock^18^. Countries included in each economic development group, energy availability, and estimates of dietary greenhouse gas emissions are reproduced (with permission) in Supplementary Table 1.

### Emissions per kilometre

We multiplied estimates of the additional energy expenditure required for a kilometre of travel by walking and cycling, with emissions per calorie to give the associated dietary-related GHG emissions.

### Uncertainty around emissions

We incorporate uncertainty around estimates of calorie availability, MET values, and dietary GHG emissions. Table 1 displays the distributions selected around each of the input parameters. Uncertainty intervals were calculated from 10,000 iterations of a Monte Carlo analysis that allowed the key parameters to vary randomly according to their pre-specified probability distributions. All calculations were done in Microsoft Excel; the uncertainty analysis was conducted using Ersatz, a specialised plug-in^27^.

**Table 1:** Distributions around key parameters used in the analysis.

Parameter	Distribution	Value
Country-level energy availability	Normal	Mean: Reported value, SD: 5% of the reported value
MET value for walking*	Normal	Mean: 3, SD: 0.6
MET value for cycling*	Normal	Mean: 3.5, SD: 0.7
MET value for inactive time*	Normal	Mean: 1.5, SD: 0.3
Walking speed	Normal	Mean: 4 kph, SD: 0.8
Cycling speed	Normal	Mean: 8.9 kph, SD: 1.78
Dietary greenhouse gas emissions**	Normal	Mean: Reported value, SD: 5% of the reported value

MET: Metabolic equivalent of task.

SD: Standard deviation.

*We assumed the SD was 20% of the mean value and then checked that the resulting distributions corresponded with the range expected from the MET values associated with these activities.

**We assumed correlated uncertainty at the study level between greenhouse gas emissions and energy intake.

### BMI and emissions under partial compensation

We explored the likely extent of compensation of energy intake Data to estimate the extent of energy compensation was drawn from a UK cohort study examining the longitudinal impact of active transport^10^. This study was selected as it provided the starting BMI and average ‘dose’ of active transport that enabled us to estimate the levels of compensation associated with observed BMI changes. Martin *et al*. observed a −0.59 (95% CI −1.11 to −0.06) change in BMI for individuals who switched from commuting by motor vehicle to commuting by active transport. The population studied had a starting BMI of 26.1, and reported a 13.9 min/day commute time after switching to active transport^10^.

We estimated the extent of compensation of energy expenditure associated with observed BMI changes using Hall *et al*.’s estimate that an energy deficit of 100 kJ (24 kcal) per day results in 1 kg of weight loss, with 50% of the weight change achieved in the first year and 95% achieved in 3 years^28^. We assumed an average height of 1.69 m (the average height in England^29^).

## Supplementary information

Supplementary Information.

## Data Availability

The dataset(s) supporting the conclusions of this article are included within the article and its Supplementary Materials files.
