# Supplementary material for: Fuelling walking and cycling: human powered locomotion is associated with non-negligible greenhouse gas emissions
Source: Sci Rep. 2020 Jun 8;10:9196. doi: 10.1038/s41598-020-66170-y (PMC7280492; doi:10.1038/s41598-020-66170-y)
Supplement: Supplementary file 1 — Supplementary Information. [file 41598_2020_66170_MOESM1_ESM.pdf]

Supplementary Information

Fuelling walking and cycling: human powered locomotion is associated with non-negligible greenhouse gas emissions

Authors: Anja Mizdrak, Linda J Cobiac, Christine L Cleghorn, Alistair Woodward, Tony Blakely

Table S1. Estimates of energy availability and dietary greenhouse gas emissions for groups of countries with similar levels of economic development, calculated from Tilman and Clark (2014) with permission

Economic group	Included countries	Energy availability (kcal/day)	Dietary greenhouse gas emissions ($kgCO_2e$ /capita/day)*	Emissions per 100 kcal ($kgCO_2e$ /100kcal)
A	Australia, Austria, Canada, Denmark, Finland, France, Germany, Ireland, Japan, The Netherlands, Norway, Sweden, Switzerland, United Kingdom, United States	3,630	11.51	0.317
B	Argentina, Chile, Greece, Israel, Italy, Malaysia, Mauritius, New Zealand, Portugal, Saudi Arabia, South Korea, Spain, Trinidad and Tobago, Uruguay, Venezuela	3,393	8.88	0.262
C	Botswana, Brazil, Colombia, Costa Rica, Ecuador, Guatemala, Iran, Jordan, Mexico, South Africa, Syria, Thailand, Tunisia, Turkey	3,266	7.03	0.215
China	China	2,769	3.44	0.124
D	Algeria, Bolivia, Cuba, Dominican Republic, Egypt, El Salvador, Indonesia, Jamaica, Lebanon, Morocco, Paraguay, Peru, Philippines, Sri Lanka, Swaziland	3,001	3.91	0.130
E Less India	Bangladesh and Pakistan, Benin, Cameroon, Cote d'Ivoire, Ghana, Honduras, Libya, Mozambique, Myanmar, Nicaragua, Nigeria, North Korea, Senegal, Vietnam	2,688	2.71	0.101
F	Burkina Faso, Central African Republic, Chad, Democratic Republic of the Congo, Eritrea and Ethiopia, Gambia, Guinea, Haiti, Kenya, Madagascar, Malawi, Zambia and Zimbabwe, Mali, Nepal, Niger, Rwanda and Burundi, Sierra Leone, Sudan (former), Tanzania, Togo, Uganda	2,309	2.43	0.105
India	India	2,296	2.43	0.106
Global average	All above	2,844	4.83	0.170

*Emissions include CO_2, N_2O, and CH_4

Table S2. Estimated excess energy expenditure required per kilometre of walking and cycling. Walking was assumed to have a MET value of 3 and a speed of 2.5mph; cycling was assigned a MET value of 4 and a speed of 5.5mph.

	Estimated excess energy expenditure (kcal/km)	
Economic group	Walking	Cycling
A	76	40
B	71	37
C	68	36
China	58	30
D	63	33
E Less India	56	29
F	48	25
India	48	25
Global average	59	31
